# Maximizing the second-harmonic generation response *via* coordination-induced localization of nonbonding electrons

**DOI:** 10.1039/d5sc06905j

**Published:** 2025-11-17

**Authors:** Jia-Xiang Zhang, A-Lan Xu, Yang Chi, Xin-Tao Wu, Hua Lin, Qi-Long Zhu

**Affiliations:** a State Key Laboratory of Structural Chemistry, Fujian Institute of Research on the Structure of Matter, Chinese Academy of Sciences Fuzhou 350002 China linhua@fjirsm.ac.cn qlzhu@fjirsm.ac.cn; b Fujian Science & Technology Innovation Laboratory for Optoelectronic Information of China Fuzhou Fujian 350108 China; c School of Materials Science and Engineering, Peking University Beijing 100871 China; d School of Chemistry and Chemical Engineering, Qufu Normal University Qufu 273165 China yang.chi@hotmail.com

## Abstract

To enhance laser frequency conversion efficiency, the development of nonlinear optical (NLO) crystals with strong second-harmonic generation (SHG) responses remains a central challenge. However, for chalcogenides, atomic-level crystal design has seldom yielded materials with SHG responses exceeding 3 × AgGaS_2_. Previous studies suggest that modulation of nonbonding electrons can enhance both linear and NLO properties, yet strategies to localize nonbonding electrons for maximizing SHG remain underexplored. Here, we demonstrate that reducing the coordination number increases the spatial localization of nonbonding electrons, thereby boosting SHG performance. Guided by this principle, we synthesized KBiP_2_S_6_ (*P*2_1_, no. 4), exhibiting the highest SHG response among sulfides to date (15 × AgGaS_2_). Atomic space tessellating analysis reveals that ∼75% of the SHG contribution originates from S, particularly from localized S-3p nonbonding electrons—challenging conventional stereochemically active lone-pair (SCALP)-based contribution models that overlook the dominant role of S. Moreover, symmetry analysis identifies the polar screw axis as the favorable symmetry for high-SHG SCALP-based chalcogenides. This work transitions NLO material design from structural building-unit assembly to electronic-level engineering, opening new avenues for next-generation high-performance NLO materials.

## Abstract 2

By leveraging coordination-induced modulation to enhance nonbonding-electron localization, KBiP₂S₆ achieves 15 times of AgGaS₂ SHG response, benefiting from the advantageous 2₁-screw-axis alignment of its SCALP units.

## Graphical abstract

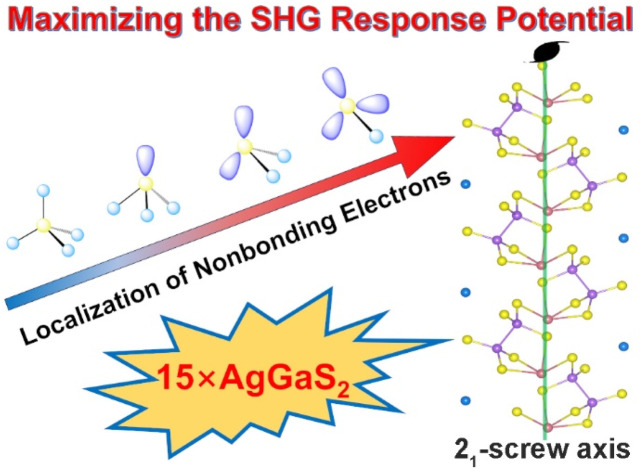

## Introduction

Second-harmonic generation (SHG), as one of the most prominent and widely applied nonlinear optical (NLO) effects, plays a critical role in laser frequency conversion.^1–3^ Among the various factors influencing the frequency doubling process, conversion efficiency is paramount. The efficiency of SHG is largely governed by two key parameters: the magnitude of the effective nonlinear coefficient (*d*_eff_) and the ability to achieve phase matching (PM). These parameters directly determine whether a crystal can deliver efficient frequency-doubled laser output. Consequently, in the field of NLO crystals, there is an ongoing pursuit of materials that exhibit strong SHG responses while maintaining a balanced set of properties suitable for practical applications.

In the infrared (IR) region, one of the most critical challenges in the study of NLO chalcogenide crystals lies in the inherent trade-off between the SHG response and band gap.^4–6^ Crystals based on selenides and tellurides often exhibit strong SHG responses, but their practical applications are severely limited by their narrow band gaps, which lead to two-photon absorption and low laser-induced damage threshold. Consequently, in the more promising sulfide-based systems, the central question that continues to attract intense research interest is: how to maximize the SHG response while avoiding a narrow band gap? This challenge is equally relevant in the ultraviolet-visible (UV-Vis) and deep-ultraviolet (DUV) regions. Previous studies have primarily focused on nonbonding electrons, where their role was analyzed within the context of symmetry. It was found that in polar tetrahedral units, the nonbonding electrons on anions exhibit favorable cooperative alignment under polar screw axes and 4̄ axis symmetry. This alignment not only enhances the SHG response but also allows for modulation of the material's linear optical properties. This strategy led to the discovery of Mg_2_PO_4_Cl,^7^ a phosphate crystal with the strongest SHG response among DUV non-π-conjugated systems, and [Ba_4_Cl_2_][CdGa_4_S_10_],^8^ a Cd-based infrared material exhibiting optimal overall performance. However, the full potential of nonbonding electrons in enhancing NLO properties remains far from fully realized.

In tetrahedral units, sulfur atoms often adopt multiple coordination environments due to corner-sharing connections, transitioning from four-fold to three-fold, two-fold, or even mono-coordination.^9^ A decrease in coordination number leads to the degree of localization of nonbonding electrons on sulfur. The increased presence of nonbonding electrons near the valence band maximum (VBM) is favorable for enhancing the compound's SHG response.^10^ Therefore, maximizing the localization of nonbonding electrons on sulfur can fully activate the deformability of S atoms, increase their polarizability under external electromagnetic fields, and ultimately enable the material to exhibit a strong SHG effect.

To maximize the localization of nonbonding electrons on sulfur, the following aspects are considered as primary goals in crystal design: (1) coordination number: to ensure rational connectivity between structural units while maintaining the maximum density and localization of nonbonding electrons on S, sulfur atoms should predominantly adopt twofold coordination within the crystal structure. (2) Interstitial cations: the incorporation of interstitial cations offers an effective strategy to disrupt dense anionic frameworks, thereby increasing structural diversity. Moreover, the formation of highly ionic bonds associated with these cations can further enhance the localization of nonbonding electrons, potentially amplifying the material's NLO response. (3) Building unit composition: in the IR region, commonly used structural units include polar tetrahedral units, stereochemically active lone-pair (SCALP) units, and other distorted polyhedra. A framework composed solely of slightly distorted polar tetrahedra tends to form various corner-sharing modes, which not only results in a high number of interconnected units but also makes it difficult to maintain predominantly two-fold-coordinated sulfur atoms. The introduction of distorted polyhedra, which connect *via* face-sharing in addition to corner- or edge-sharing, is also suboptimal. In contrast, SCALP units, characterized by large distortion and a tendency to connect *via* corner-sharing while spatially separating from each other, provide a promising alternative.^11^ Therefore, a rational design strategy can be realized by constructing frameworks centered on SCALP units bridged by tetrahedral units. The next step involves the selection of appropriate elements from the periodic table. Previous studies have demonstrated that within a given group, increased covalence of the M–S bond reduces the deformability of nonbonding electrons. As such, Pb^2+^ and Bi^3+^, which are heavy metals with strong lone-pair activity, are preferred as central atoms in SCALP units.^12^ To ensure a sufficiently wide band gap, light elements from the second and third periods—such as Li^+^, Mg^2+^, Al^3+^, Si^4+^, and P^5+^—are considered as auxiliary cations. Excluding those likely to form six-fold coordination, only Si^4+^ and P^5+^ remain.^13^

After integrating the aforementioned crystal design principles, we refocused our research on the ABiP_2_S_6_ system, which had not been fully understood previously.^14,15^ Earlier experiments demonstrated that RbBiP_2_S_6_ exhibited the highest SHG response among sulfides at the time (11.9 × benchmark AgGaS_2_).^14^ However, under the traditional framework of anionic group theory, the deep connection between the high SHG response and the complex inorganic structure, especially in systems with significant multi-atom cooperative interactions, remained unclear. To overcome this limitation, we shifted our focus from a “group-centered” to an “atom-centered” perspective and quantitatively analyzed the SHG contributions of each atom in the anionic group. This analysis revealed that, in KBiP_2_S_6_, approximately 75% of the SHG response originates from the coordinated sulfur atoms, rather than the traditionally emphasized central atom. This finding challenges the previous design paradigm, which placed primary focus on the central atom, and emphasizes the importance of modulating the local coordination environment of the Q^2−^ anion through the central atom to enhance its polarizability. Guided by this revised structure–property relationship, we optimized the experimental measurements and successfully synthesized millimeter-level KBiP_2_S_6_ crystals, which exhibited the highest SHG response among known sulfides (15 × benchmark AgGaS_2_). This value significantly exceeds its previously reported performance,^15^ improving by a factor of 8.3. Not only does this result validate the effectiveness of our theoretical model, but it also suggests that the potential performance of some previously reported crystals has yet to be fully explored. To extend the applicability of this design strategy, we further statistically analyzed the spatial configurations and symmetry features of SCALP units. We found that polar helical axes, as an advantageous symmetry, can provide a strong theoretical basis for early-stage screening, aiding the structure prediction and exploration of high-performance NLO materials.

## Results and discussion

**Fig. 1:**
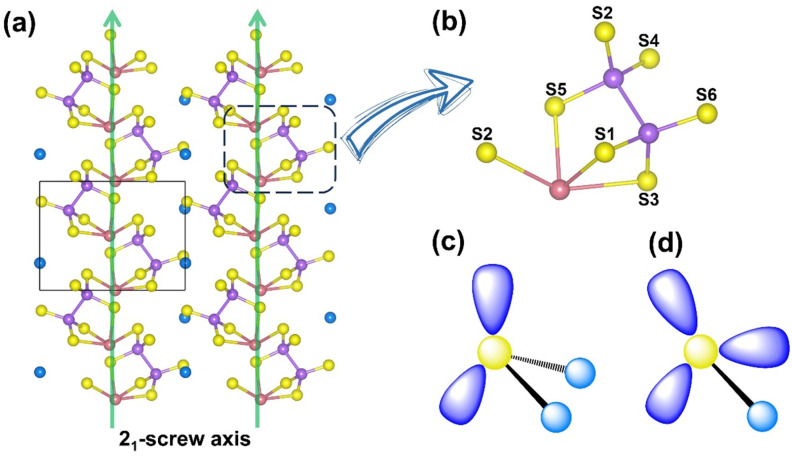
The structure of KBiP_2_S_6_: (a) the chain-like structure arranged around the 2_1_-screw axis; (b) the minimum repeating units [BiP_2_S_7_], which are composed of [BiS_4_] and [P_2_S_6_]; the sketch of (c) two-coordinated S at S1, S2, S3, and S5 and (d) mono-coordinated S at S4 and S6.

KBiP_2_S_6_ crystallizes in the non-centrosymmetric space group *P*2_1_ (Table S1), with its high-symmetry axis aligned along the *b*-axis in the monoclinic system, corresponding to the direction of the 2_1_ screw axis. Due to the primitive lattice (P-lattice), the unit cell lacks atoms at body-centered and face-centered positions, and all atoms occupy general Wyckoff positions (2*a*), indicating the absence of high-symmetry crystallographic sites (Table S2). To simplify the coordination description of Bi, the strongly bonded Bi–S interactions (<3.0 Å) were modeled as discrete [BiS_4_] units (Tables S3 and S4). These [BiS_4_] units connect with [P_2_S_6_] groups *via* both corner-sharing and edge-sharing modes, forming composite [BiP_2_S_7_] units. These composite units further connect through corner-sharing along the 2_1_-screw axis, generating one-dimensional chain-like structures (Fig. 1). Interestingly, the distortion orientation of the [BiS_4_] units along the local *C*_2_ axis is consistent with the direction of the 2_1_-screw axis in the crystal, resulting in a uniform alignment of the polar [BiS_4_] units along the polar axis. This directional consistency favors constructive SHG response accumulation. Within this chain structure, most S atoms are two-fold coordinated, particularly those shared between [BiS_4_] and [P_2_S_6_] units. However, S4 and S6 atoms from the [P_2_S_6_] group exhibit mono-coordination, extending outward from the chains. This structural arrangement closely aligns with the original crystal design strategy, and the coherent alignment of polar units along the polar axis provides a robust structural foundation for the observed strong SHG response.

**Fig. 2:**
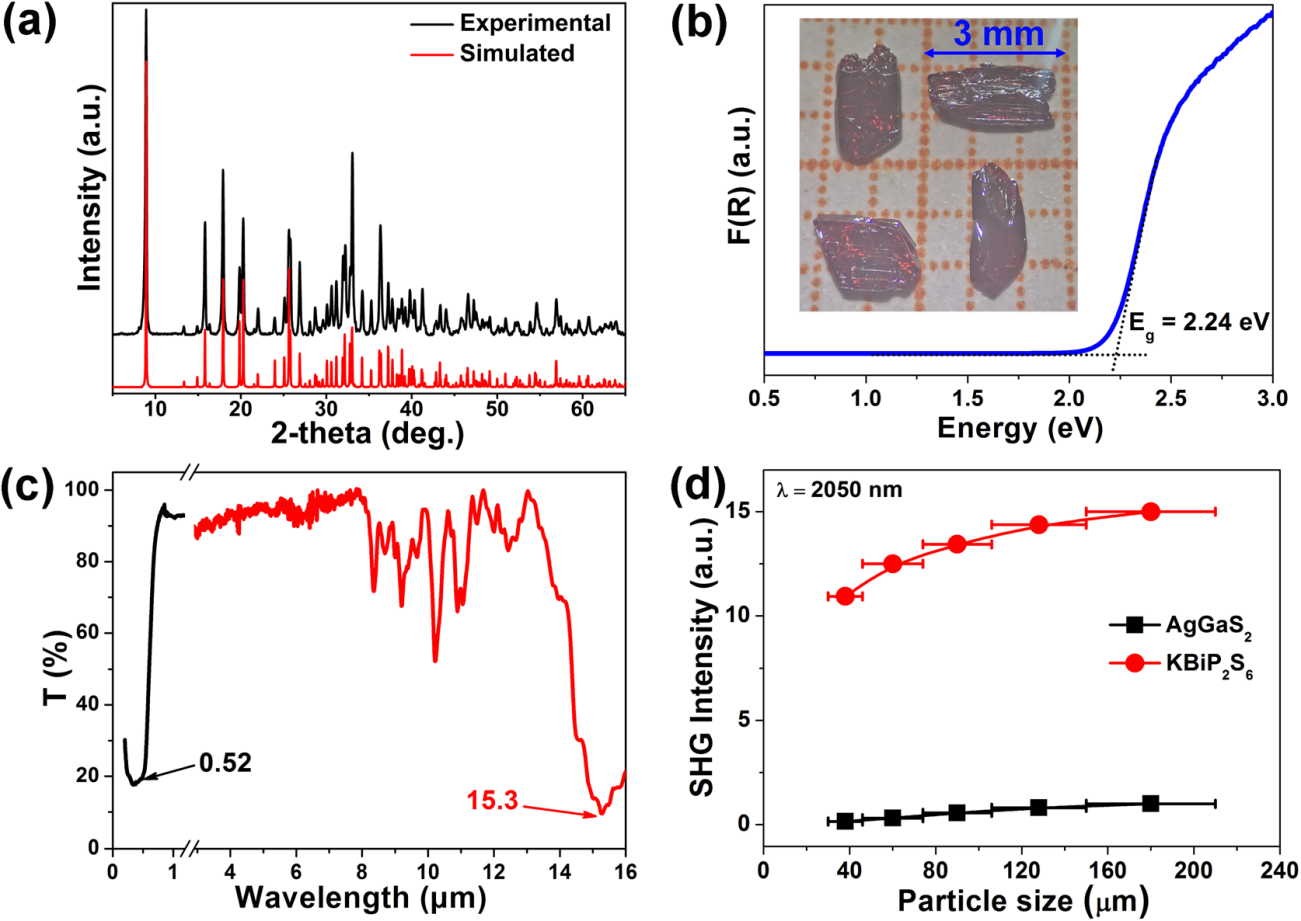
The characterization of KBiP_2_S_6_: (a) pure sample XRD pattern of KBiP_2_S_6_; (b) *E*_g_ of 2.24 eV with the corresponding 3 mm red block crystal; (c) optical transparent range from 0.52–15.3 µm and (d) plots of powder SHG response *versus* particle size.

Energy-dispersive X-ray spectroscopy (EDX) (Fig. S1) and powder X-ray diffraction (XRD) (Fig. 2a) confirmed the phase purity of the compound. Thermal analysis indicated that the compound begins to decompose at 800 K (Fig. S2). Further XRD analysis of the thermally treated sample revealed significant changes in peak intensities and the appearance of new diffraction peaks, supporting the thermal decomposition result (Fig. S3). Diffuse reflectance spectroscopy performed on powder samples showed an optical band gap (*E*_g_) of 2.24 eV, consistent with the observed red color of the crystals (Fig. 2b). Furthermore, the LIDT of KBiP_2_S_6_ was evaluated employing the single-pulse method,^16^ yielding a value of 27.64 MW cm^−2^ at 1064 nm. This result is significantly higher than that of the benchmark material AgGaS_2_ (2.82 MW cm^−2^) measured under the same conditions.^17^ IR transmission measurements on the single crystal, in conjunction with the reflectance data, revealed a broad transmission window ranging from 0.52 to 15.3 µm (Fig. 2c). It is widely recognized that higher crystallinity correlates with fewer intrinsic defects, thereby more accurately reflecting a material's intrinsic optical properties. Our previous study on RbBiP_2_S_6_ revealed an SHG response as high as 11.9 times that of AgGaS_2_,^14^ whereas other reports on its isostructural counterpart, KBiP_2_S_6_,^15^ reported an SHG response of only 1.8 times AgGaS_2_. This significant disparity in performance, arising solely from the substitution of alkali metal cations, contradicts the widely accepted anionic group theory, which attributes NLO properties primarily to the behavior of anionic structural units. In previous studies, the direct synthesis of KBiP_2_S_6_ crystals from elemental precursors often resulted in limited crystal quality and size, primarily due to the constraints of solid–solid reactions.^15^ To overcome this challenge, we adopted an optimized synthesis route utilizing a boron-chalcogen synthesis method,^18^ with KI added as a flux agent to improve both reaction activity and crystal quality. Powder SHG measurements employing the Kurtz–Perry technique were conducted to evaluate the SHG coefficient of KBiP_2_S_6_. Fig. 2d demonstrates a distinct particle-size dependence of SHG intensity for KBiP_2_S_6_, where enhanced SHG responses with increasing particle size suggest phase-matching behavior. Remarkably, at a laser wavelength of 2050 nm, KBiP_2_S_6_ exhibits an SHG efficiency 15 times greater than that of AgGaS_2_ within the particle size range of 150–210 µm. These results underscore the importance of evaluating crystallinity as a key factor in SHG measurements, especially for NLO crystal candidates. Furthermore, this insight suggests that previously reported NLO crystals with excellent structural features may exhibit even better performance if optimized for higher crystallinity. Moreover, a distinctive testing result was observed in the SHG measurements of KBiP_2_S_6_. Across the particle size range from small to large, the crystal consistently exhibited a strong SHG response. This behavior differs significantly from that of most compounds, which typically show a pronounced increase in SHG intensity with increasing particle size, eventually reaching a plateau. Calculation of the coherence length^19^ revealed a value of only 4 µm, which is smaller than the initial particle sizes used in the tests. As a result, in the highly crystalline sample, the SHG response showed no significant increase with particle size. This combination of a short coherence length and a large SHG response offers promising potential for further investigation of KBiP_2_S_6_ in ultrafast optics and SHG microscopy applications.

**Fig. 3:**
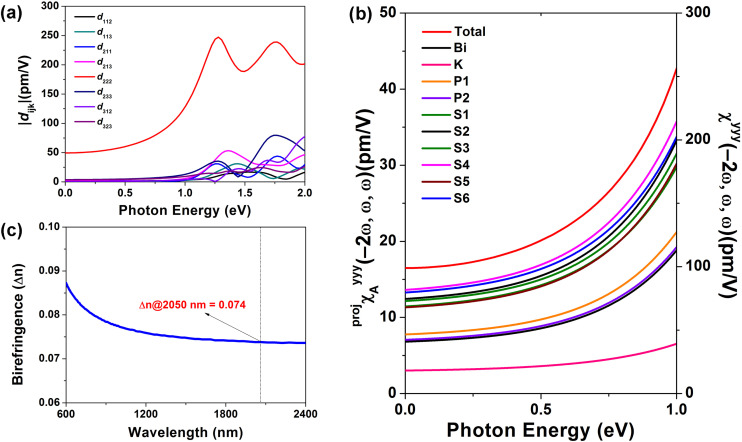
The linear and NLO calculation analysis of KBiP_2_S_6_: (a) all eight non-zero *d*_*ijk*_ tensors, (b) the birefringence (Δ*n*) at 2050 nm is 0.074 and (c) the second-order NLO polarizability and its projection onto different atoms at different energies of the photoelectric field.

The structure-based predictions and experimental results confirm that the structure–property relationship of this compound aligns well with current theoretical models. To further investigate the individual contributions of each structural unit, we performed a detailed SHG response analysis of the compound using the AST method,^20^ which is particularly suitable for evaluating contributions in systems containing SCALP units. Since KBiP_2_S_6_ crystallizes in the low-symmetry 2 point group, the Kleinman symmetry,^21^ which is often used to simplify NLO tensor analysis, can significantly deviate in low-symmetry compounds. Therefore, to ensure accuracy, all eight non-zero second-order NLO coefficients for this compound were calculated (Fig. 3a). In the 0–1.0 eV incident photon energy range, all coefficients except *d*_222_ have nearly identical absolute values and contribute minimally to the overall response. However, beyond 1.0 eV, significant discrepancies arise among tensor components that would be considered equivalent under Kleinman symmetry. Overall, the effective SHG coefficient (*d*_eff_) of KBiP_2_S_6_ is dominated by the *d*_222_ tensor component, and the macroscopic second-order polarization of the compound increases with rising incident photon energy (Fig. 3b). Besides, its birefringence calculation results show that it has a birefringence of 0.074 at 2050 nm, which can effectively compensate for the phase mismatch caused by dispersion (Fig. 3c).^22^

Compared to conventional static tensor analysis, the AST method offers a dynamic perspective, more accurately reflecting the induced dipole behavior of the material under an external optical field. Therefore, the contributions to the dominant *d*_222_ component were further traced. As shown in Table 1, under a 2050 nm laser field, S atoms contribute a remarkable 74.93%, whereas the combined contribution from Bi and P is only 22.19%, and K accounts for a minor 2.89%. If the analysis was conducted from the traditional functional group perspective, the contributions of the [BiS_4_] and [P_2_S_6_] units would be 30.93% and 66.19%, respectively. This comparison clearly shows that the conventional approach significantly underestimates the role of S atoms in SHG activity. Attributing the significant contribution of S atoms solely to their associated structural units tends to shift researchers' focus toward the central elements, which may lead to a misinterpretation of the overall properties of the functional unit.

**Table 1:** The contribution percentage of each atom in atomic space to the SHG susceptibility *d*_222_ obtained using the AST scheme, at an incident wavelength of 2050 nm

Atom	Contributions (%)	Atom	Contributions (%)
Bi	7.00	S2	12.61
K	2.89	S3	12.19
P1	7.95	S4	13.77
P2	7.24	S5	11.47
S1	11.59	S6	13.30

**Fig. 4:**
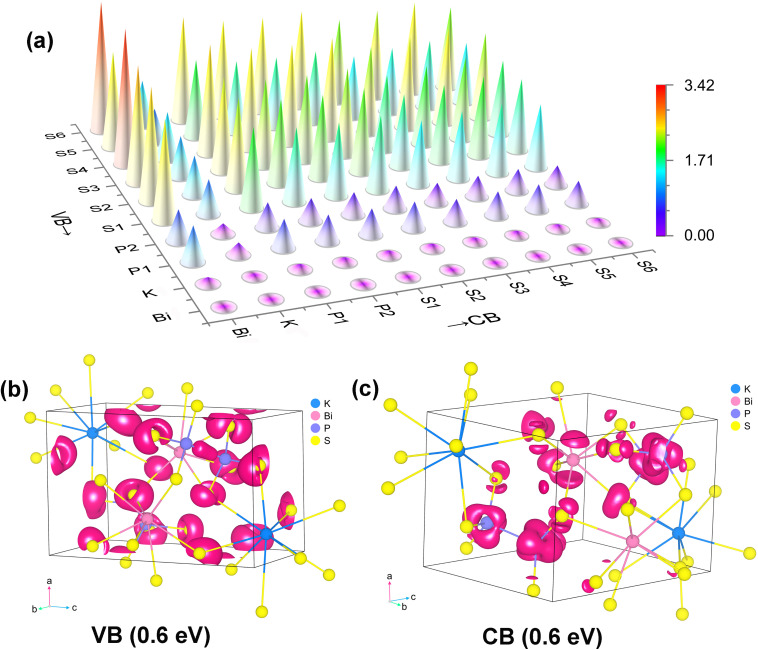
The origination analysis of SHG responses in KBiP_2_S_6_: (a) transition dipole moment component matrix diagram, and the SHG distribution functions in the (b) VB and (c) CB at 0.6 eV (2050 nm).

By visualizing the contribution of each atom in the form of diagrams, it is clearly observed that S atoms exhibit significantly greater contributions than other elements across the 0–1 eV energy range. Through analysis using atomic spatial dipole component matrix maps, it was revealed that at 2050 nm (0.6 eV), the dipole moments of all atomic species in KBiP_2_S_6_ contribute positively to the macroscopic second-order nonlinear polarization (Fig. 4a; the numerical data are provided in Table S5 in the SI). The dominant instantaneous dipole moments responsible for this response primarily arise from S–Bi, S–P, and S–S interactions, whereas transitions such as P–S, P–P, and S–K contribute to a similar but much lesser extent. This indicates that the key contributors to the SHG response are the transitions involving non-bonding electrons on S atoms concentrated near the valence band maximum (VBM). As previously discussed in the structural analysis, KBiP_2_S_6_ contains S atoms with two different coordination environments: two-coordinated S1, S2, S3, and S5, and mono-coordinated S4 and S6. The diagram clearly shows that the most significant contributions to the polarization originate from transitions involving S4 and S6 to Bi. Additionally, transitions from S4 and S6 to K, P, and other sulfur atoms also exceed those of the two-coordinated sulfur atoms. In order to visualize the contributions of atoms in their VB and CB, further atomic SHG contributions were visualized at 0.6 eV (Fig. 4b and c).

**Fig. 5:**
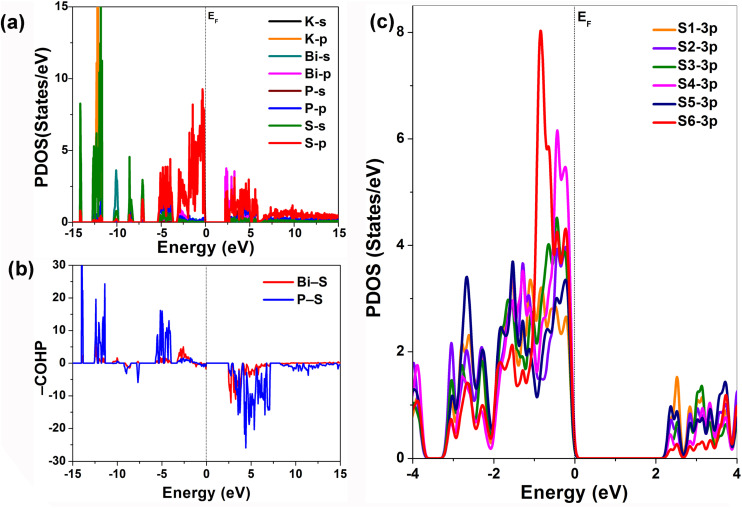
The SHG contribution analysis in SCALP units: (a) the PDOS of KBiP_2_S_6_, which shows the almost S-3p contribution in the VBM; (b) the COHP analysis of total Bi–S and P–S bonds; (c) S-3p states of two-coordinate S atoms (S4 and S6) and the mono-coordinate S atoms (S1–S3 and S5).

In addition to the contribution analysis at 0.6 eV, visualizations were also conducted at 0 eV and 1 eV (Fig. S4 and S5). Combined with the projected density of states (PDOS), it is evident that the VB is primarily composed of S-3p and P-3p orbitals, while the conduction band (CB) is mainly derived from Bi-6p and S-3p orbitals (Fig. 5a). This aligns well with the previously discussed transition contributions, confirming that the VBM is dominated by non-bonding S-3p states. Further supporting this, the PDOS curves of the two-coordinate S atoms (S4 and S6) and the mono-coordinate S atoms (S1–S3 and S5) clearly show that the S-3p states of the mono-coordinate atoms possess higher non-bonding electron states near the VBM compared to the two-coordinate atoms (Fig. 5c). This also strongly corroborates the view that mono-coordinate S atoms have more non-bonding states, thereby inducing a larger microscopic contribution to the SHG (Fig. 4a and Table 1). In oxide-based systems,^23–26^ it has been suggested that the greater the activity of the s^2^ lone pair electrons on SCALP-type metal cations, the more significant their contribution to SHG. In the case of KBiP_2_S_6_, the VBM is not solely composed of Bi-6s orbitals but also includes contributions from Bi-6p orbitals.

When evaluating the s-orbital activity using the *R*_sca_ factor,^23^ a value of 2.6 is obtained. If one were to overlook the differences in bonding strength between sulfides and oxides and directly apply the correlation between higher *R*_sca_ values and stronger SHG responses, then Bi in KBiP_2_S_6_ would be expected to contribute significantly to SHG. This appears to coincide with our experimental observations. However, PDOS calculations clearly demonstrate that the VBM in KBiP_2_S_6_ is overwhelmingly dominated by non-bonding S-3p states, and that the contributions from Bi-6s and Bi-6p orbitals, as well as from their antibonding interactions with S, are negligible. In our earlier study of RbBiP_2_S_6_, static *d*_222_ tensor calculations showed that the VB-I region (−2.3 to 0 eV) plays a crucial role in the SHG response. This energy range corresponds to a region in KBiP_2_S_6_ where non-bonding S-3p states are highly concentrated and where nonbonding interactions between S and Bi/P also reside (Fig. 5b and S6). A similar conclusion has also been observed in nitrides.^27^ Cheng *et al.*^27^ reported that in the MP_2_N_4_ (M = Ge, Sn, and Pb) series, the positive contribution of nonbonding states to the SHG response increases along the Ge-to-Pb substitution. Notably, only the Pb-based compound avoids the occurrence of negative SHG contributions that typically arise from overly strong bonding interactions, highlighting the beneficial role of weakly bound or nonbonding electrons in enhancing NLO performance. Evidently, the above contradictory result highlights the fundamental differences in bonding strength between oxide and chalcogenide systems, underscoring the inadequacy of treating SCALP building units as unified contributors in chalcogenides. The AST-based analysis of the oxide compound CsSbF_2_SO_4_ has shown that, under both static fields and incident light at 1064 nm, the contribution of Sb significantly decreases.^20^ This reduction is attributed to changes in the dipole moment associated with certain O–Sb transitions, which become oppositely aligned with the macroscopic second-order nonlinear polarization vector as the photon energy increases.

In contrast, such behavior is not observed in the sulfide KBiP_2_S_6_, providing further evidence that conclusions derived from oxide-based SCALP systems may not directly apply to their chalcogenide counterparts. In particular, the difference in atomic radii between oxygen and sulfur plays a critical role. The smaller radius of oxygen in oxides typically leads to structural units exhibiting SOJT distortions in octahedral environments with 3-, 4-, or 5-coordination.^28^ However, in sulfide systems, the larger atomic radius of sulfur introduces greater variability in bond lengths and coordination geometries, leading to distinct structural and electronic responses that must be considered independently from those of oxides.

Analysis of the Bi–S bond lengths in the [BiS_4_] unit reveals that the shorter Bi–S1 and Bi–S5 bonds (2.713 and 2.726 Å) experience stronger bonding interactions compared to the longer Bi–S2 and Bi–S3 bonds (2.806 and 2.898 Å). This indicates a higher degree of covalency in the Bi–S1 and Bi–S5 bonds, which in turn leads to a reduced delocalization of the non-bonding electron density on S1 and S5. Consequently, S1 and S5 contribute less to the SHG response than S2 and S3, which could be observed in S-3p PDOS. Within the [P_2_S_6_] unit, the strong covalent nature of the internal P–S bonds results in a relatively narrow bond length distribution (2.030–2.071 Å) for P–S1, S2, S3, and S5, indicating minimal variation in bonding strength. Even the bond lengths involving the singly coordinated sulfur atoms S4 and S6 are only slightly shortened to 1.988 and 1.967 Å, respectively. These subtle changes are insufficient to significantly strengthen the binding of the non-bonding electrons on S4 and S6, thereby allowing a higher degree of nonbonding electron localization. As a result, S4 and S6 exhibit markedly stronger SHG contributions. Compared to their bi-coordinated counterparts, mono-coordinated S atoms possess more nonbonding valence electrons. This results in higher chemical activity and enhanced deformability of their electron clouds under external optical fields. Physically, the *d*_222_ tensor component corresponds to the interaction of two optical electric fields polarized along the *b*-axis with the crystal, generating a nonlinear dipole oscillation that is also aligned with the *b* direction. Consequently, a stronger alignment of an S atom's electron cloud with the *b*-axis leads to a greater induced polarization. In KBiP_2_S_6_, the electron clouds of both mono- and bi-coordinated S atoms are predominantly oriented along the *b* direction, which explains the structure's large SHG response. Furthermore, differences in bond lengths and angles due to the distinct bonding environments of S inevitably alter their electron cloud orientations, thereby leading to their distinct contributions to the overall SHG effect. These structure–property correlation insights, supported by theoretical calculations, validate our design strategy centered on increasing the localization degree of nonbonding electrons on sulfur atoms. S atoms with lower coordination numbers are shown to contribute more significantly to the SHG response. Furthermore, this analysis underscores the limitations of treating the SCALP building unit as a unified contributor in chalcogenides, suggesting that a more nuanced approach is required for accurate evaluation of NLO performance.

**Fig. 6:**
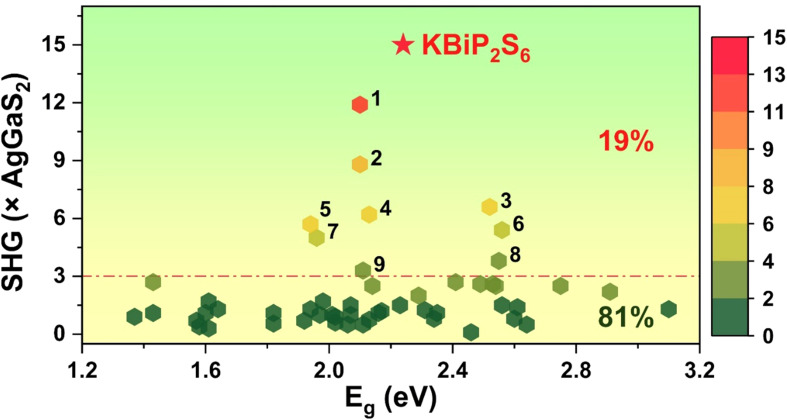
The statistical analysis of 53 SCALP-based chalcogenide compounds. The dashed line shows the SHG responses of 3 times AgGaS_2_. (1) RbBiP_2_S_6_, (2) Hg_3_AsSe_4_I, (3) Na_6_Sn_3_P_4_S_16_, (4) Hg_3_AsSe_4_Br, (5) SbSI, (6) NaPb_3_P_4_S_16_, (7) PbGa_2_GeSe_6_, (8) SnGa_4_Se_7_ and (9) PbGa_4_Se_7_.

A statistical analysis of the currently known 53 PM SCALP-based chalcogenides (Table S6) shows that they can be categorized by the group of the central SCALP atom—namely groups 14, 15, and 16 (Fig. 6). Using a SHG efficiency of 3 × AgGaS_2_ as a performance threshold, only 10 compounds (19%) surpass this value, including PbGa_4_Se_7_ (*Pc*, 3.3 × AgGaS_2_),^29^ SnGa_4_Se_7_ (*Pc*, 3.8 × AgGaS_2_),^30^ PbGa_2_GeSe_6_ (*Fdd*2, 5 × AgGaS_2_),^31^ NaPb_3_P_4_S_16_ (*R*3*m*, 5.4 × AgGaS_2_),^32^ SbSI (*Pna*2_1_, 5.7 × AgGaS_2_),^33^ Hg_3_AsSe_4_Br (*P*6_3_*mc*, 6.2 × AgGaS_2_),^34^ Na_6_Sn_3_P_4_S_16_ (*R*3*m*, 6.6 × AgGaS_2_),^35^ Hg_3_AsSe_4_I (*P*6_3_*mc*, 8.8 × AgGaS_2_),^34^ RbBiP_2_S_6_ (*P*2_1_, 11.9 × AgGaS_2_)^14^ and KBiP_2_S_6_ (*P*2_1_, 15 × AgGaS_2_). Except for SbSI, the remaining nine compounds are all constructed by combining tetrahedral units with SCALP units. In our previous studies, we identified that tetrahedral units preferentially adopt symmetry elements such as polar screw axes and fourfold roto-inversion axes. This raises the question of whether SCALP units also exhibit similar symmetry preferences. Structural analysis of these nine high-performance SHG compounds reveals that, except for SnGa_4_Se_7_ and PbGa_4_Se_7_, which crystallize in the *Pc* space group, the others all crystallize in polar space groups containing screw axes: *Pna*2_1_ (2_1_), *Fdd*2 (2_1_), *R*3*m* (3_1_ or 3_2_), and *P*6_3_*mc* (6_3_). For SCALP units possessing SOJT activity, distortions along the *C*_2_, *C*_3_, or *C*_4_ axes reduce the original *O*_h_ symmetry of ideal octahedra to lower point groups such as *D*_2d_, *C*_3v_, or *C*_4v_. Further distortion from ideal configurations arises due to variations in coordination environments. Because of the significant distortion inherent to SCALP units, their crystallization in non-centrosymmetric space groups typically gives rise to a net dipole moment, externally manifesting as structures in polar space groups. These space groups often contain rotational axes and mirror planes, which—combined with translational symmetry—form screw axes and glide planes. The presence of screw axes implies an axial alignment of SCALP units within the crystal, which can occur in two forms: (1) the central element of the SCALP unit occupies a symmetry site on the screw axis, yielding a standard unit geometry with *D*_2d_, *C*_3v_, or *C*_4v_ symmetry. The corresponding screw axes are 2-, 3- (or 6_3_-), and 4-fold, and this scenario ensures consistent stacking along the axis; (2) the SCALP units are helically arranged around the screw axis, leading to distorted geometries. In this case, the uniformity in stacking is reflected by the axial components of their dipole moments. Naturally, due to the flexible coordination number of SCALP units, their coordination environments are not limited to strong 3-, 4-, or 5-coordination; weaker bonding interactions may result in higher coordination numbers. Therefore, achieving advantageous alignment of SCALP units within crystals will require further exploration of how different structural units induce orientation preferences in SCALP motifs. This constitutes a key direction for future research.

## Conclusions

In this work, we revisited the ABiP_2_S_6_ system, which had not been fully understood in the context of previous crystal design principles. RbBiP_2_S_6_, previously reported as having the highest SHG response among sulfides (11.9 × AgGaS_2_), exhibited a strong response but lacked a deep structural explanation under the conventional anionic group theory, particularly in systems with significant multi-atom cooperation. To overcome this limitation, we shifted from a “group-centered” to an “atom-centered” approach, quantitatively analyzing the SHG contributions of each atom within the anionic groups. Our findings revealed that in KBiP_2_S_6_, approximately 75% of the SHG response originates from the coordinated sulfur atoms rather than the central atom traditionally emphasized. This challenges the previous design focus on central atoms and underscores the importance of controlling the coordination environment of Q^2−^ anions to enhance their polarizability. Based on this structure–property relationship, we synthesized millimeter-level KBiP_2_S_6_ crystals, which exhibit the highest SHG response reported for sulfides to date (15 × AgGaS_2_), an *E*_g_ of 2.24 eV, a high LIDT (9.8 × AgGaS_2_ @1064 nm), a wide transmission window ranging from 0.52 to 15.3 µm, and a Δ*n* of 0.074. This not only validates the proposed design model but also suggests that the full potential of some previously reported crystals may still be unexplored. Additionally, by analyzing the spatial configurations and symmetry features of SCALP units, we identified polar helical axes as a promising symmetry motif, providing a theoretical tool for early-stage screening and discovery of high-performance NLO materials.

## Author contributions

Jia-Xiang Zhang: investigation, formal analysis, writing – original draft. A-Lan Xu: formal analysis. Yang Chi: methodology, validation, writing – review & editing. Xin-Tao Wu: conceptualization, writing – review & editing. Hua Lin: supervision, conceptualization, writing – review & editing. Qi-Long Zhu: supervision, writing – review & editing.

## Conflicts of interest

There are no conflicts to declare.

## Supplementary Material

SC-OLF-D5SC06905J-s001

SC-OLF-D5SC06905J-s002

## Data Availability

CCDC 2153282 contains the supplementary crystallographic data for this paper.^36^ All supplementary data for the results of this study are available in the article and its supplementary information (SI). Supplementary information: additional experimental and theoretical results, together with additional tables and figures. See DOI: https://doi.org/10.1039/d5sc06905j.
