# Supplementary material for: Maximizing the second-harmonic generation response via coordination-induced localization of nonbonding electrons
Source: Chem Sci. 2025 Nov 17;17(2):1249–58. doi: 10.1039/d5sc06905j (PMC12641426; doi:10.1039/d5sc06905j)
Supplement: SC-017-D5SC06905J-s001 [file SC-017-D5SC06905J-s001.pdf]

Supplementary Information (SI) for Chemical Science.
This journal is © The Royal Society of Chemistry 2025

Electronic Supplementary Information (ESI)

Maximizing the Second-Harmonic Generation Response via Coordination-Induced Localization of Nonbonding Electrons

Jia-Xiang Zhang,[a,b,c] A-Lan Xu,[a,b] Yang Chi,*[,d] Xin-Tao Wu,[a,b] Hua Lin,*[,a,b] and Qi-Long Zhu*[,a,b]

[a]*State Key Laboratory of Structural Chemistry, Fujian Institute of Research on the Structure of Matter, Chinese Academy of Sciences, Fuzhou 350002, China*

[b]*Fujian Science & Technology Innovation Laboratory for Optoelectronic Information of China, Fuzhou 350108, China*

[c]*School of Materials Science and Engineering, Peking University, Beijing 100871, China*

[d]*School of Chemistry and Chemical Engineering, Qufu Normal University, Qufu 273165, China*

*Corresponding authors: yang.chi@hotmail.com, linhua@fjirsm.ac.cn and qlzhu@fjirsm.ac.cn.

Electronic Supplementary Information (ESI)

Contents

1 Experimental Section 6

Figure S1. Characterizations of $KBiP_2S_6$: (a) EDX results and (b) SEM image and corresponding elemental mapping analysis. 11

Figure S2. Thermal test results of $KBiP_2S_6$...12

Figure S3. XRD test results after TG of $KBiP_2S_6$. 12

Figure S4. Origination analysis of SHG responses in $KBiP_2S_6$: (a) transition dipole moment component matrix diagram, and the SHG distribution functions in (b) VB and (c) CB at 0 eV..13

Figure S5. Origination analysis of SHG responses in $KBiP_2S_6$: (a) transition dipole moment component matrix diagram, and the SHG distribution functions in (b) VB and (c) CB at 1 eV..14

Figure S6. Crystal orbital Hamilton population (COHP) analysis of the (a) Bi–S and (b) P–S bonds. 15

Figure S7. Comparison of the electronic band structures of $KBiP_2S_6$ (a) without and (b) with spin-orbit coupling (SOC) interaction...15

Figure S8. PDOS of $KBiP_2S_6$ with spin-orbit coupling (SOC) interaction................16

Figure S9. Comparison of the d_{222} tensor component of $KBiP_2S_6$ (a) with and (b) without spin-orbit coupling (SOC) interaction. 16

Table S1. Crystal data and structure refinement for $KBiP_2S_6$. 17

Table S2. Fractional atomic coordinates ($\times10^4$) and equivalent isotropic displacement parameters ($Å^2\times10^3$) for $KBiP_2S_6$. 18

Table S3. Bond lengths for $KBiP_2S_6$. 18

Table S4. Bond angles (deg.) for $KBiP_2S_6$. 19

Table S5. The original data used to generate the plot in Figure 4a. 20

Table S6. A summary of reported PM SCALP-based chalcogenide IR-NLO materials. 21

Electronic Supplementary Information (ESI)

1 Experimental Section

1.1 Reagents

The following reactant was stored into the Ar-filled glovebox (oxygen and moisture levels was less than 0.1 ppm). Bi_2O_3 (99.9%, Aladdin), B (99.9%, Aladdin), S (99.9%, Aladdin), P (99%, ABCR), and KI (99.99%, Aladdin) were used to synthesis.

1.2 Synthesis

Polycrystalline sample of $KBiP_2S_6$ was prepared using a high-temperature solid-state reaction. After numerous explorations on the experimental conditions including starting reactant, loading ratio, annealing temperature, the optimal synthesis condition was established as loading a stoichiometry mixture (about 500 mg in total) of Bi_2O_3, B, S, P, and KI in a molar ratio of 1:2:12:4:1 in a fused-silica tube under vacuum. Then, the tubes were subjected to crystallization in a computer-controlled muffle furnace. The temperature was ramped up to 973 K needed over 100 hours, held at this temperature for 60 hours, and then gradually reduced to 673 K at a rate of 2 K/h. After the furnace was switched off, the samples were allowed to cool naturally to room temperature. The synthesized crystals remained stable in air and moisture for over half year.

1.3 Crystal Structure Determinations

Choosing some high-quality crystals of $KBiP_2S_6$ with suitable sizes were selected for single-crystal X-ray diffraction (SCXRD) analysis. The single-crystal diffraction data collections were collected by using graphite-monochromatized Mo Kα radiation (λ = 0.7107 Å) at 100 K on a Rigaku XtaLAB Synergy-R diffractometers install with a

Hybrid Pixel Array Detector [1]. The crystal structure of $KBiP_2S_6$ was solved by using the ShelXT, and then refined by the ShelXL on Olex2 package [2]. The program PLATON was used to check for the structure. Crystallographic data and structure refinements of $KBiP_2S_6$ were given in the Table S1. Atomic coordinates, equivalent isotropic displacement parameters, anisotropic displacement parameters, selected bond angles and lengths for $KBiP_2S_6$ were shown in Tables S2-S4.

1.4 Powder X-ray diffraction

Powder X-ray diffraction (PXRD) analysis was carried out in a Rigaku Mini-Flex II powder diffractometer (Cu-Kα, λ = 1.5418 Å) at room temperature in the angular range of 2θ = 5–65° with a scan step size of 0.02°.

1.5 Energy-dispersive X-ray spectroscopy

The semi-quantitative energy dispersive X-ray spectrometer (EDX, Oxford INCA) were measured with a field emission scanning electron microscope (FESEM, JSM6700F).

1.6 UV-vis-NIR Diffuse Reflectance spectroscopy

UV-vis-NIR absorption measurement was performed in the region of 190−2500 nm at room temperature using an UV-vis-NIR spectrometer (Perkin-Elmer Lambda 950). The reflectance spectrum of the $BaSO_4$ powder was collected as the baseline and the diffuse reflectance data were converted to absorbance internally by the instrument by use of the Kubelka-Munk function [3].

Electronic Supplementary Information (ESI)

1.7 Thermal Analysis

The thermal stability analyses were measured on a NETZSCH STA 449C simultaneous analyzer. Thermogravimetric (TG) analysis and differential thermal analysis (DTA) were measured by Netzsch STA 499C installation. The samples about 10.0 mg were placed in alumina crucibles and heated in 300–1123K under N_2 atmosphere.

1.8 Second-Harmonic Generation (SHG) Measurements

According to Kurtz-Perry method [4], the powder SHG property measurement was performed in 2050 nm Q-switch laser radiation (10 mJ). $AgGaS_2$ was taken as a contrast material in the same condition. The different granule size scopes (30−46, 46−74, 74−106, 106−150, 150−210 μm) of $KBiP_2S_6$ and $AgGaS_2$ were ground and screen out. The SHG intensity of the frequency-doubled output gave out the prepared materials were observed via a photomultiplier tube and saved on the oscilloscope.

1.9 Laser Induced Damage Threshold (LIDT) Measurements

The LIDT of $KBiP_2S_6$ was carried out single pulse measurement method[5] and similar scope of $AgGaS_2$ single crystal used to the reference. The whole measuring materials were foisted into selfsame plastic holders (thickness: 1 mm and diameter: 8 mm), respectively. Using an optical microscope monitor the exterior change of sample under the 1064 nm laser radiation with pulse width τ_p of 10 ns. Nova II sensor with a PE50-DIF-C energy sensor and a Vernier caliper was used for measuring the power of laser beam and the damage spot radius.

Electronic Supplementary Information (ESI)

2 Computational Details

Electronic structure calculation of $KBiP_2S_6$ was carried out within the framework of density functional theory (DFT) by using the *CASTEP* package[6]. The generalized gradient approximation (GGA) within the Perdew-Burke-Ernzerhof (PBE)-type exchange-correlation potentials were used throughout this work[7]. The employed OTFG norm-conserving pseudopotentials of K, Bi, P, and S treat $3s^2$ $3p^6$ $4s^1$, $6s^2$ $6p^3$, $3s^2$ $3p^3$, and $3s^2$ $3p^4$ as the valence states, respectively. The plane-wave cutoff energy of 480 eV and the threshold of 5×10^{-7} eV/atom were set for the self-consistent-field convergence of the total electronic energy. The atomic positions were allowed to relax to minimize the internal forces. An excellent convergence of the energy differences (5.0×10^{-6} eV/atom), maximum force (0.01 eV/Å), and maximum displacement (5.0×10^{-4} Å) was implemented in the atomic position optimization. A $4 \times 3 \times 3$ Monkhorst-Pack *k*-point grid in the Brillouin Zone of the primitive cell is chosen and more than 240 empty bands were involved in the calculations to ensure the convergence of SHG coefficients. Scissors implementation within length-gauge formulations of the frequency dependent NLO response equations used to calculate the second-order response function were originally derived by Aversa and Sipe and later modified by F. Nastos et al[8,9]. To show the interactions between different elements, the Crystal Orbital Hamilton Populations (COHP)[10] curves of selected interactions in $KBiP_2S_6$ were carried out by using the tight-binding linear muffin-tin orbital (TB-LMTO) method with atomic spheres approximation (ASA).[11]

It is well recognized that calculations incorporating spin-orbit coupling (SOC)

can provide a more accurate description of band dispersion, particularly in systems containing heavy elements. To evaluate the specific influence of SOC in the present compound, we have performed additional calculations of the band structure and PDOS with SOC included (see Figures S7 and S8). In Figure S7, the lowered conduction band does contain significant contributions from Bi 6p_1/2. However, the PDOS reveals that the contribution of S to the CBM is not negligible. The combined contributions of S 3p_1/2 and S 3p_3/2 are not significantly weaker than Bi 6p_1/2 (Figure S8). Moreover, the distribution here does not differ much from the PDOS obtained without considering the SOC effect (Figure 5a). Additionally, it should be emphasized that the inclusion of SOC breaks the independence of spin and orbital angular momentum. However, the changes in the orbital composition of the relevant energy bands are not significant, and the difference in the calculated SHG coefficients is also small (see Figure S9). Therefore, based on the minor impact of SOC on orbital composition and SHG coefficients observed here, the computational results without SOC were adopted to enable a more streamlined analysis of SHG contributions.

Electronic Supplementary Information (ESI)

3. Figures and Tables

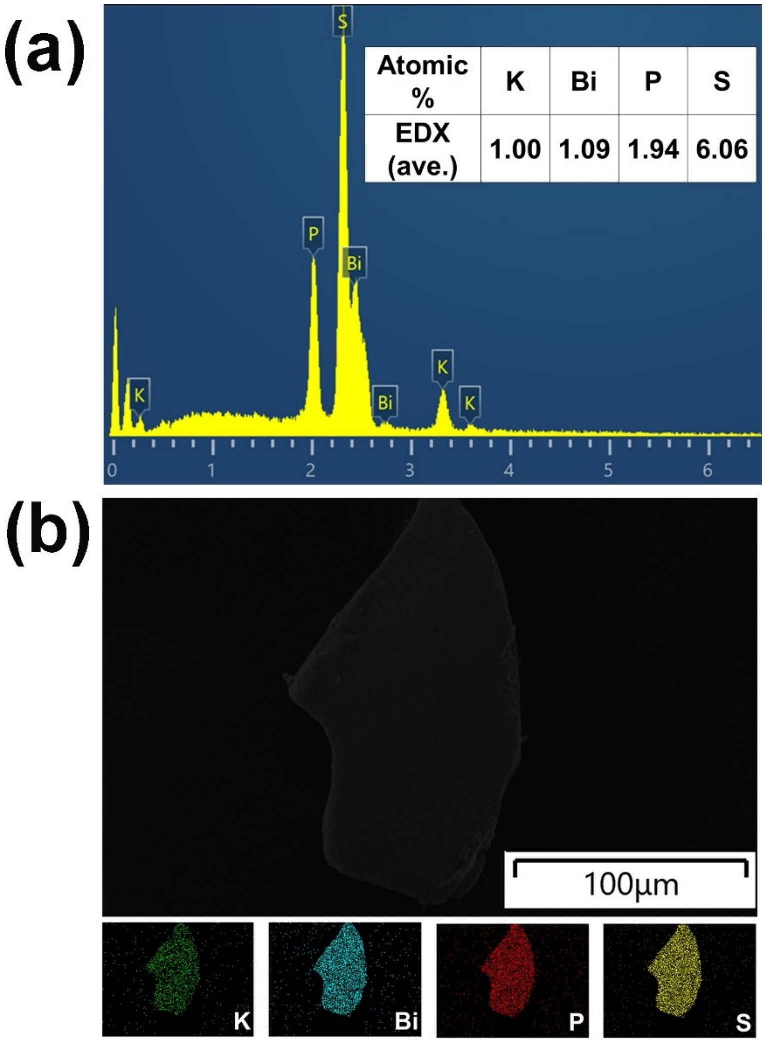

Figure S1. Characterizations of $KBiP_2S_6$: (a) EDX results and (b) SEM image and corresponding elemental mapping analysis.

Electronic Supplementary Information (ESI)

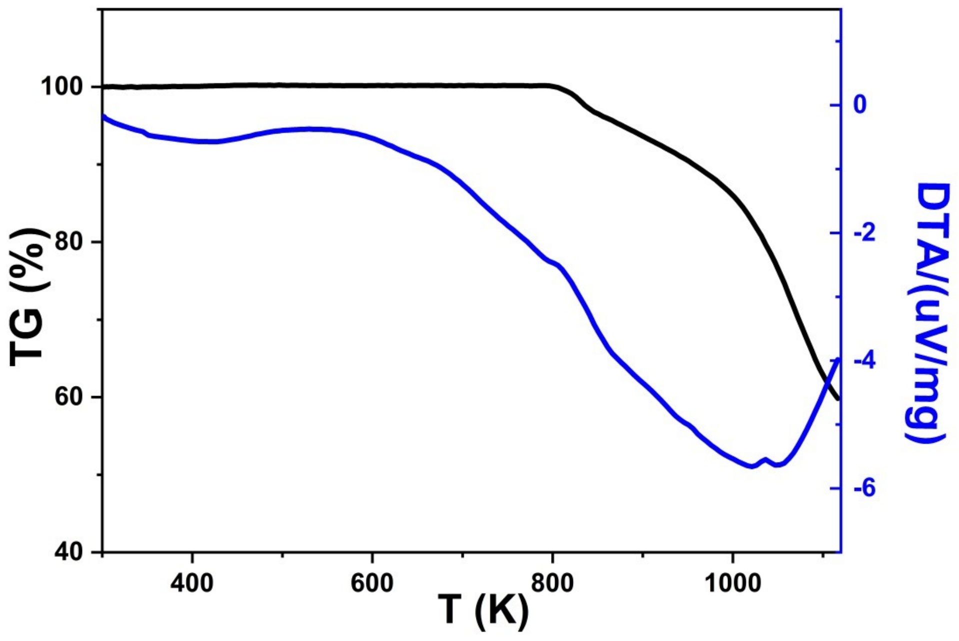

Figure S2. Thermal test results of $KBiP_2S_6$.

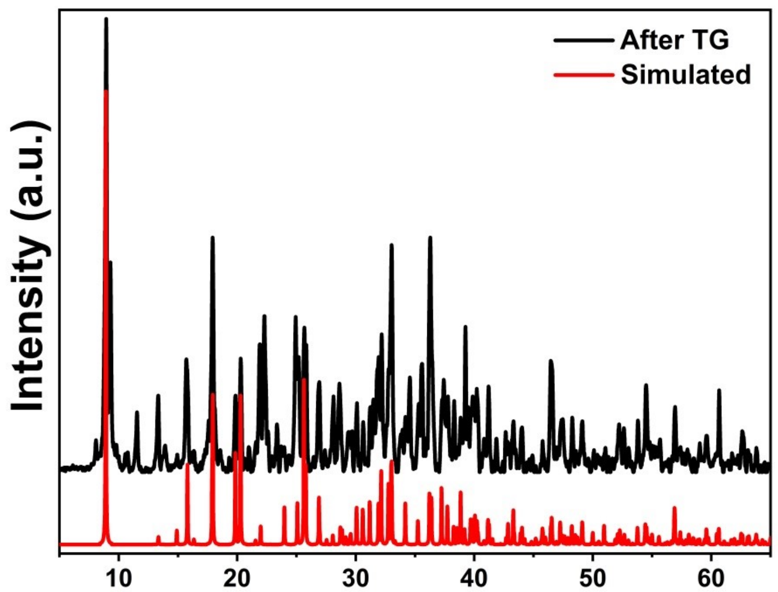

Figure S3. XRD test results after TG of $KBiP_2S_6$.

Electronic Supplementary Information (ESI)

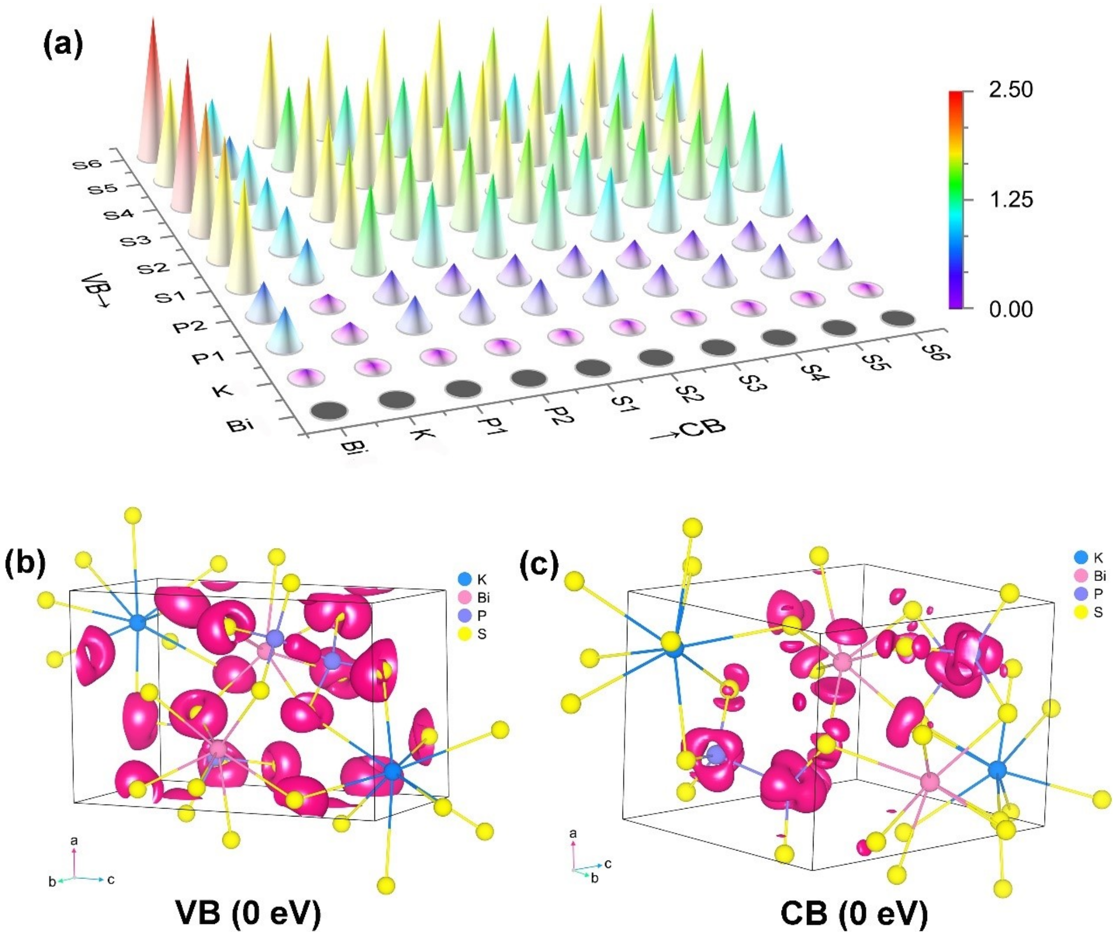

Figure S4. Origination analysis of SHG responses in $KBiP_2S_6$: (a) transition dipole moment component matrix diagram, and the SHG distribution functions in (b) VB and (c) CB at 0 eV.

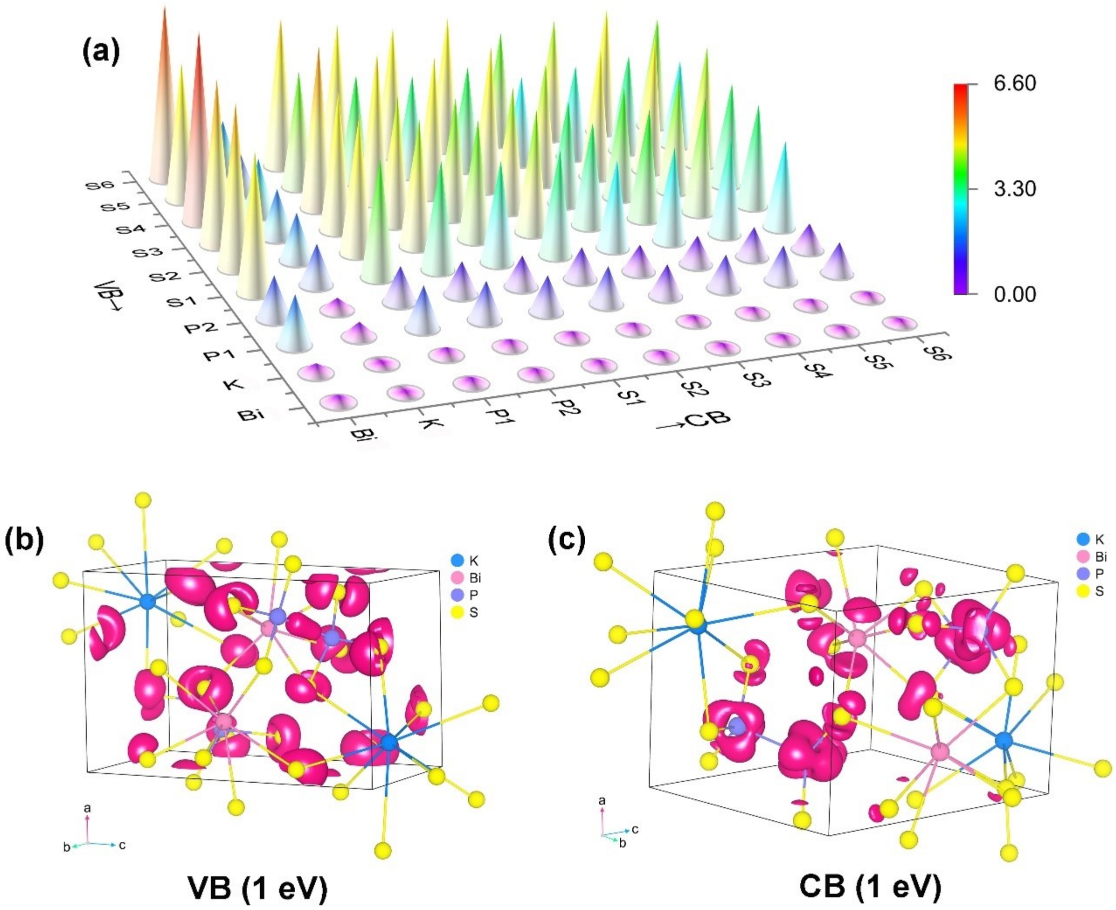

Figure S5. Origination analysis of SHG responses in $KBiP_2S_6$: (a) transition dipole moment component matrix diagram, and the SHG distribution functions in (b) VB and (c) CB at 1 eV.

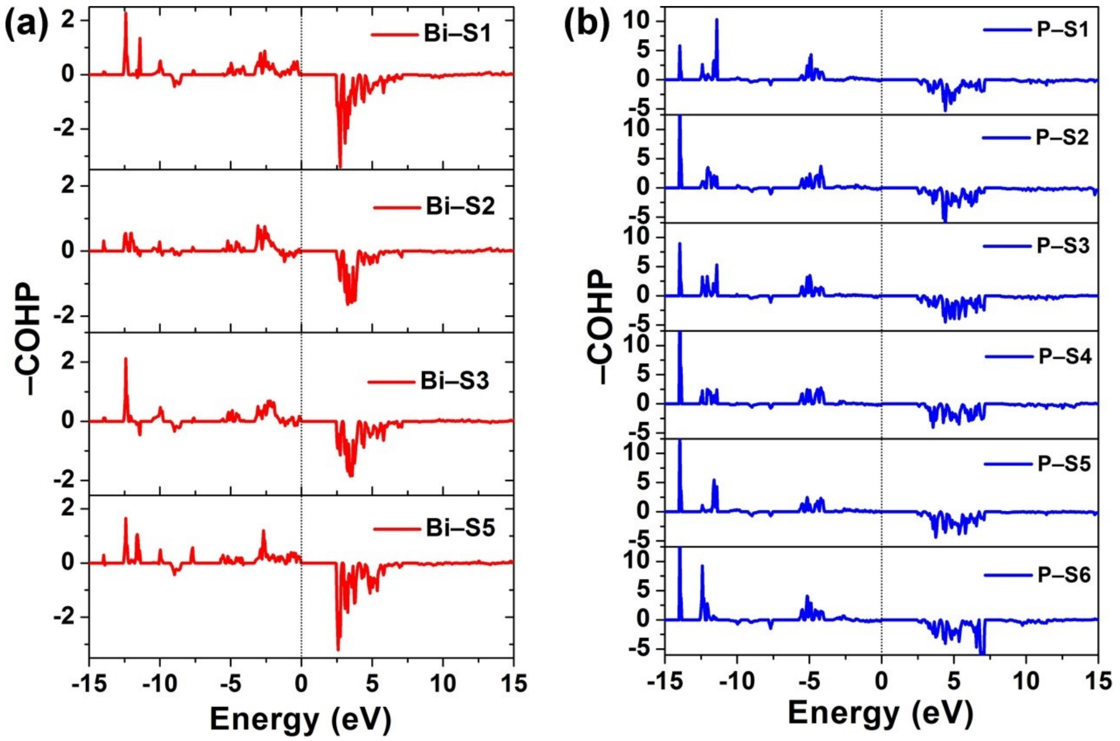

Figure S6. Crystal orbital Hamilton population (COHP) analysis of the (a) Bi–S and (b) P–S bonds.

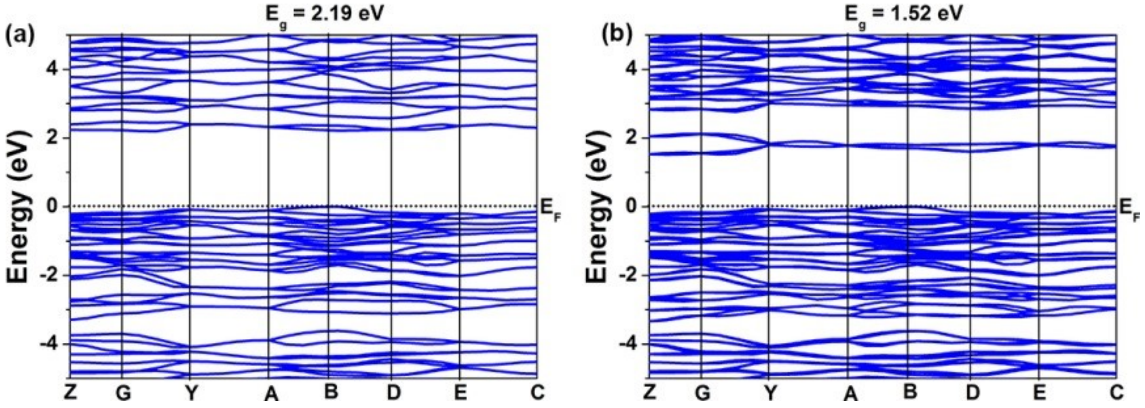

Figure S7. Comparison of the electronic band structures of $KBiP_2S_6$ (a) without and (b) with spin-orbit coupling (SOC) interaction.

Electronic Supplementary Information (ESI)

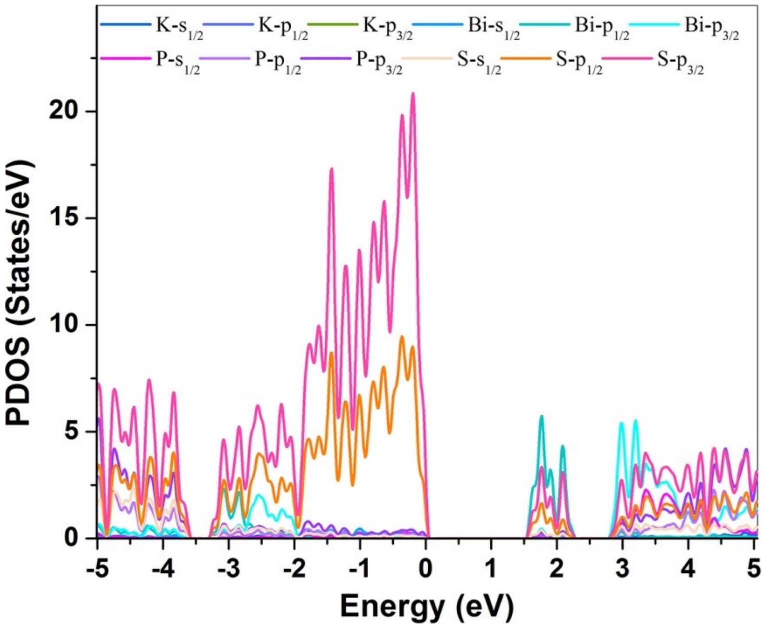

Figure S8. PDOS of $KBiP_2S_6$ with spin-orbit coupling (SOC) interaction.

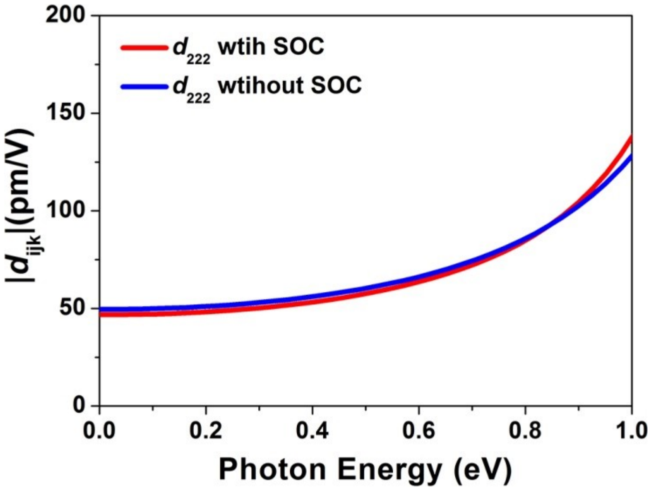

Figure S9. Comparison of the d_{222} tensor component of $KBiP_2S_6$ (a) with and (b) without spin-orbit coupling (SOC) interaction.

Electronic Supplementary Information (ESI)

Table S1. Crystal data and structure refinement for $KBiP_2S_6$.

Molecular formula	$KBiP_2S_6$
CCDC number	2153282
Formula Weight	502.38
Crystal system	monoclinic
Space group	$P2_1$
Temperature(K)	293(2)
Radiation	Mo K_α (λ = 0.71073)
F(000)	456
a(Å)	6.629(3)
b(Å)	7.416(3)
c(Å)	9.919(5)
β(deg.)	92.097(8)
V(Å^3)	487.3(4)
Z	2
$\rho_{cal.}$ (g/cm^3)	3.424
μ (mm^{-1})	20.054
GOOF on F^2	1.092
Flack factor	–0.028(10)
R_1,wR_2 [$I > 2\sigma(I)$]	0.0373, 0.0863
R_1, wR_2 (all data)	0.0391, 0.0872
Largest diff. peak and hole (e·Å^{-3})	1.58/–2.36

Electronic Supplementary Information (ESI)

Table S2. Fractional atomic coordinates (×10^4) and equivalent isotropic displacement parameters (Å^2×10^3) for $KBiP_2S_6$.

Atom	*Wyckoff*	x	y	z	U_{eq} (Å)*
K	2*a*	2024(5)	7501(7)	21(4)	31.2(10)
Bi	2*a*	2938.6(7)	0.0(14)	4802.4(5)	25.1(3)
P1	2*a*	1874(6)	4608(6)	3257(4)	20.4(11)
P2	2*a*	2957(6)	2328(6)	2000(4)	18.8(9)
S1	2*a*	519(6)	1500(6)	3062(4)	22.3(9)
S2	2*a*	4138(6)	6476(6)	3254(4)	21.3(9)
S3	2*a*	846(6)	404(6)	2311(4)	22.7(10)
S4	2*a*	748(6)	462(5)	7545(4)	22.1(10)
S5	2*a*	1599(7)	3440(7)	5138(4)	21.9(9)
S6	2*a*	3229(7)	3137(7)	127(4)	27.3(10)

*U_{eq} is defined as 1/3 of the trace of the orthogonalized U_{ij} tensor.

Table S3. Bond lengths for $KBiP_2S_6$.

Bond	Length (Å)	Bond	Length (Å)
P1–S2	2.043(6)	Bi–S1	2.713(4)
P1–S4	1.989(6)	Bi–S2	2.898(4)
P1–S5	2.071(6)	Bi–S3	2.806(4)
P2–S1	2.059(6)	Bi–S5	2.726(5)
P2–S1	2.030(6)	P1–P2	2.235(6)
P2–S3	1.967(6)		

Table S4. Bond angles (deg.) for $KBiP_2S_6$.

Bond angle	degree	Bond angle	degree
S1-Bi-S2	81.18(12)	S2-P1-S5	112.0(3)
S1-Bi-S3	72.45(12)	S4-P1-P2	108.2(2)
S1-Bi-S5	84.83(13)	S4-P1-S2	114.5(3)
S3-Bi-S2	147.44(12)	S4-P1-S5	112.9(2)
S5-Bi-S2	77.18(13)	S5-P1-P2	103.1(2)
S5-Bi-S3	81.66(13)	S1-P2-P1	102.5(2)
S2-P1-P2	105.1(2)	S3-P2-P1	102.1(2)
S6-P2-P1	118.1(3)	S6-P2-S3	116.4(3)
P2-S1-Bi	85.42(18)	P1-S2-Bi	102.5(2)

Table S5. The original data used to generate the plot in Figure 4a.

		→CB									
		**Bi1**	**K1**	**P1**	**P2**	**S1**	**S2**	**S3**	**S4**	**S5**	**S6**
↑ VB	**Bi1**	0.0009	0.0003	0.0008	0.0007	0.0007	0.0007	0.0006	0.0007	0.0007	0.0006
	**K1**	0.0066	0.0023	0.0054	0.0050	0.0050	0.0051	0.0044	0.0047	0.0049	0.0044
	**P1**	0.0341	0.0120	0.0279	0.0258	0.0255	0.0261	0.0226	0.0240	0.0251	0.0227
	**P2**	0.0299	0.0106	0.0244	0.0226	0.0224	0.0229	0.0198	0.0210	0.0220	0.0199
	**S1**	0.0959	0.0339	0.0784	0.0725	0.0718	0.0734	0.0636	0.0675	0.0705	0.0638
	**S2**	0.1095	0.0387	0.0896	0.0828	0.0820	0.0838	0.0726	0.0770	0.0805	0.0729
	**S3**	0.1139	0.0402	0.0932	0.0861	0.0853	0.0871	0.0755	0.0801	0.0837	0.0758
	**S4**	0.1335	0.0471	0.1091	0.1009	0.0999	0.1021	0.0884	0.0939	0.0981	0.0888
	**S5**	0.0956	0.0338	0.0782	0.0723	0.0716	0.0731	0.0633	0.0672	0.0703	0.0636
	**S6**	0.1303	0.0460	0.1065	0.0985	0.0975	0.0997	0.0863	0.0916	0.0958	0.0867

Table S6. A summary of reported PM SCALP-based chalcogenide IR-NLO materials.

Number	Formula	Crystallographic parameters		Optical properties				Ref.
		Unit Cell	Space Group	E_g (eV)[a]	SHG response (× $AgGaS_2$)[b]	Δn[c]	PM	
★	**$KBiP_2S_6$**	**Monoclinic**	***$P2_1$***	**2.24**	**15**	**0.074@2050 nm**	**Yes**	**This work**
1	$RbBiP_2S_6$	Monoclinic	*$P2_1$*	2.1	11.9	0.061@2050 nm	Yes	[12]
2	Hg_3AsSe_4I	Hexagonal	*$P6_3mc$*	2.10	8.8	0.36@2050 nm	Yes	[13]
3	$Na_6Sn_3P_4S_{16}$	Trigonal	*R3m*	2.52	6.6	0.12@2090 nm	Yes	[14]
4	Hg_3AsSe_4Br	Hexagonal	*$P6_3mc$*	2.13	6.2	0.37@2050 nm	Yes	[13]
5	SbSI	Orthorhombic	*$Pna2_1$*	1.94	5.7	0.62@1910 nm	Yes	[15]
6	$NaPb_3P_4S_{16}$	Trigonal	*R3m*	2.56	5.4	0.102@2090 nm	Yes	[16]
7	$PbGa_2GeSe_6$	Orthorhombic	*Fdd2*	1.96	5	0.114@2050 nm	Yes	[17]
8	$SnGa_4Se_7$	Monoclinic	*Pc*	2.55	3.8	N/A	Yes	[18]

9	$PbGa_4Se_7$	Monoclinic	*Pc*	2.11	3.3	0.08@1910 nm	Yes	[19]
10	$Ba_2As_2Se_5$	Monoclinic	$P2_1$	1.43	2.7	N/A	Yes	[20]
11	$[K_2PbI][Ga_7S_{12}]$	Orthorhombic	*Imm*2	2.41	2.7	N/A	Yes	[21]
12	$[Na_2PbI][Ga_7S_{12}]$	Orthorhombic	*Imm*2	2.53	2.6	0.069@1910 nm	Yes	[22]
13	$[K_2PbBr][Ga_7S_{12}]$	Orthorhombic	*Imm*2	2.49	2.6	N/A	Yes	[21]
14	$[K_2PbCl][Ga_7S_{12}]$	Orthorhombic	*Imm*2	2.54	2.5	N/A	Yes	[21]
15	$RbPbPS_4$	Orthorhombic	$P2_12_12_1$	2.75	2.5	0.109@2900 nm	Yes	[23]
16	$Sn_2Ga_2S_5$	Orthorhombic	$Pna2_1$	2.14	2.5	0.25@2050 nm	Yes	[24]
17	$KSbP_2S_6$	Monoclinic	$P2_1$	2.91	2.2	N/A	Yes	[25]
18	$Ba_7Sn_5S_{15}$	Hexagonal	$P6_3cm$	2.29	2	N/A	Yes	[26]
19	KSb_5S_8	Monoclinic	*Pn*	1.61	1.7	0.289@1910 nm	Yes	[27]
20	$SnGa_2GeSe_6$	Orthorhombic	*Fdd*2	1.98	1.7	N/A	Yes	[28]
21	$Sn_7Br_{10}S_2$	Hexagonal	$P6_3$	2.56	1.5	0.116@2100 nm	Yes	[29]

22	La_4InSbS_9	Tetragonal	$P4_12_12$	2.07	1.5	N/A	Yes	[30]
23	$K_2Ag_3Sb_3S_7$	Orthorhombic	$Cmc2_1$	2.23	1.5	0.165@2050 nm	Yes	[31]
24	$Pb_2P_2S_6$	Monoclinic	Pn	2.61	1.4	N/A	Yes	[32]
25	Pb_4SeBr_6	Orthorhombic	$Imm2$	1.94	1.3	0.127@2050 nm	Yes	[33]
26	$SnGa_4S_7$	Monoclinic	Pc	3.1	1.3	N/A	Yes	[18]
27	$Pb_2As_2S_5$	Monoclinic	$P2_1$	1.64	1.3	0.195@2050 nm	Yes	[34]
28	$AsI_3{\cdot}(S_8)_3$	Trigonal	$R3m$	2.31	1.25	N/A	Yes	[35]
29	$SnPS_{2.86}Se_{0.14}$	Monoclinic	Pn	2.17	1.2	N/A	Yes	[36]
30	$SnPS_3$	Monoclinic	Pn	2.35	1.1	0.11@2100 nm	Yes	[36]
31	$Ba_2AsSbSe_5$	Monoclinic	$P2_1$	1.43	1.1	N/A	Yes	[20]
32	$RbMnAs_3S_6$	Trigonal	$R3$	2.16	1.1	0.16@1910 nm	Yes	[37]
33	$RbSb_5S_8$	Monoclinic	Pn	1.6	1.1	0.287@1910 nm	Yes	[27]
34	$PbSnSiS_4$	Monoclinic	$P2_1$	1.82	1.1	N/A	Yes	[38]

35	$Ba_2As_2S_5$	Orthorhombic	$Pca2_1$	1.97	1	N/A	Yes	[20]
36	$RbAg_2TeS_6$	Hexagonal	$P6_3cm$	2.04	1	N/A	Yes	[39]
37	$CsAg_2TeS_6$	Hexagonal	$P6_3cm$	2.04	1	N/A	Yes	[39]
38	$CsMnAs_3S_6$	Trigonal	$R3$	2.02	0.9	0.14@1910 nm	Yes	[37]
39	$Ba_2As_{1.25}Bi_{0.75}Se_5$	Monoclinic	$P2_1$	1.37	0.9	N/A	Yes	[20]
40	$Sn_{11}(PS_4)_4I_{10}$	Tetragonal	$I\bar{4}2d$	2.34	0.8	0.068@546 nm	Yes	[40]
41	$Pb_{3.5}GeS_4Br_3$	Hexagonal	$P6_3$	2.6	0.8	N/A	Yes	[41]
42	Sm_4InSbS_9	Tetragonal	$P4_12_12$	2.13	0.75	N/A	Yes	[42]
43	$Ba_2As_{1.5}Bi_{0.5}S_5$	Monoclinic	$P2_1$	1.57	0.7	N/A	Yes	[20]
44	$Ba_2As_2S_{4.8}Se_{0.2}$	Monoclinic	$P2_1$	1.92	0.7	N/A	Yes	[20]
45	$Cs_2Ag_3Sb_3S_7$	Orthorhombic	$Cmc2_1$	2.02	0.6	0.153@2100 nm	Yes	[43]
46	$Ba_2As_{1.25}Sb_{0.75}S_5$	Monoclinic	$P2_1$	1.82	0.55	N/A	Yes	[20]
47	$Rb_2Ag_3Sb_3S_7$	Orthorhombic	$Cmc2_1$	2.11	0.5	0.147@2100 nm	Yes	[43]

48	La_2CuSbS_5	Orthorhombic	*Ima*2	2.06	0.5	0.11@2050 nm	Yes	[44]
49	$PbGa_2GeS_6$	Orthorhombic	*Fdd*2	2.64	0.5	N/A	Yes	[45]
50	$K_3Mn_2Sb_3S_8$	Tetragonal	$I\bar{4}2m$	1.58	0.4	N/A	Yes	[46]
51	$Rb_3Mn_2Sb_3S_8$	Tetragonal	$I\bar{4}2m$	1.61	0.3	0.074@2050 nm	Yes	[46]
52	β-$PbGa_2S_4$	Orthorhombic	$Pna2_1$	2.46	0.1	0.04@1910 nm	Yes	[47]

[a]Experimental value; [b]powder sample; [c] Theoretical value.

Electronic Supplementary Information (ESI)

References

[1] CrysAlisPro, 1.171. 36.28 ed., Agilent Technologies, Santa Clara, CA, 2013.

[2] O. V. Dolomanov, L. J. Bourhis, R. J. Gildea, J. A. K. Howard and H. Puschmann, *J. Appl. Crystallogr.* 2009, **42**, 339–341.

[3] P. Kubelka and F. Munk, Z. *Tech. Physik.* 1931, **12**, 593–601.

[4] S. K. Kurtz and T. T. Perry, *J. Appl. Phys.* 1968, **39**, 3798–3813.

[5] M.-J. Zhang, X.-M. Jiang, L.-J. Zhou and G.-C. Guo, *J. Mater. Chem. C*, 2013, **1**, 4754–4760.

[6] S. J. Clark, M. D. Segall, C. J. Pickard, P. J. Hasnip, M. I. J. Probert, K. Refson and M. C. Payne, *Z. Kristallogr. - Cryst. Mater.* 2005, **220**, 567–570.

[7] J. P. Perdew, K. Burke and M. Ernzerhof, *Phys. Rev. Lett.* 1996, **77**, 3865.

[8] C. Aversa and J. E. Sipe, *Phys. Rev. B: Condens. Matter Mater. Phys.* 1995, **52**, 14636–14645.

[9] F. Nastos, B. Olejnik, K. Schwarz and J. E. Sipe, *Phys. Rev. B: Condens. Matter Mater. Phys.* 2005, **72**, 045223.

[10] R. Dronskowski and P. E. Bloechl, *J. Phys. Chem.*, 1993, **97**, 8617–8624.

[11] O. Jepsen, A. Burkhardt and O. K. Andersen, The *TB-LMTO-ASA* Program, version 4.7, Max-Planck-Institut für Festkörperforschung, Stuttgart, Germany, 1999.

[12] M.-M. Chen, S.-H. Zhou, W. Wei, M.-Y. Ran, B. Li, X.-T. Wu, H. Lin and Q.-L. Zhu, *ACS Materials Letters* 2022, **4**, 1264–1269.

[13]J. Chen, C. Lin, X. Jiang, G. Yang, M. Luo, X. Zhao, B. Li, G. Peng, N. Ye, Z. Hu, J. Wang and Y. Wu, *Mater. Horiz.* 2023, **10**, 2876–2882.

[14] C. Zhao, B. Zhang, X. Tian, G. Zhou, J. Xu and K. Wu, *Inorg. Chem. Front.* 2023, **10**, 5726–5733.

[15] Y. Zhou, L.-T. Jiang, X.-M. Jiang, B.-W. Liu and G.-C. Guo, *Chin. Chem. Lett.* 2025, **36**, 109740.

[16] C. Zhao, D. Lu, X. Tian, J. Xu, B. Zhang, K. Wu, H. Yu and H. Zhang, *Inorg. Chem.* 2023, **62**, 21487–21496.

[17] Z.-Z. Luo, C.-S. Lin, H.-H. Cui, W.-L. Zhang, H. Zhang, H. Chen, Z.-Z. He and W.-D. Cheng, *Chem. Mater.* 2015, **27**, 914–922.

[18] Z.-Z. Luo, C.-S. Lin, H.-H. Cui, W.-L. Zhang, H. Zhang, Z.-Z. He and W.-D. Cheng, *Chem. Mater.* 2014, **26**, 2743–2749.

[19] Z. X. Zheng, Z. X. Qiu, C. H. Xie, Y. P. Zhang, X. M. Jiang, B. W. Liu and G. C. Guo, *Sci. China Mater.* 2023, **66,** 2795–2802.

[20] A. K. Iyer, J. B. Cho, M. J. Waters, J. S. Cho, B. M. Oxley, J. M. Rondinelli, J. I. Jang and M. G. Kanatzidis, *Chem. Mater.* 2022, **34**, 5283–5293.

[21] W.-F. Chen, B.-W. Liu, S.-M. Pei, X.-M. Jiang and G.-C. Guo, *Adv. Sci.* 2023, **10**, 2207630.

[22] Z.-X. Wu, W.-F. Chen, X.-M. Jiang, B.-W. Liu and G.-C. Guo, *Chem. Mater.* 2024, **36**, 3444–3451.

[23] A. Y. Wang, S.-H. Zhou, M.-Y. Ran, B. Li, X.-T. Wu, H. Lin and Q.-L. Zhu, *Inorg. Chem. Front.* 2024, **11**, 3744–3754.

[24] M.-Y. Li, B. Li, H. Lin, Z. Ma, L.-M. Wu, X.-T. Wu and Q.-L. Zhu, *Chem. Mater.* 2019, **31**, 6268–6275.

[25] V. Nguyen, B. Ji, K. Wu, B. Zhang and J. Wang, *Chem. Sci.* 2022, **13**, 2640-2648.

[26] Z.-Z. Luo, C.-S. Lin, W.-D. Cheng, H. Zhang, W.-L. Zhang and Z.-Z. He, *Inorg. Chem.* 2013, **52**, 273–279.

[27] W.-F. Chen, B.-W. Liu, S.-M. Pei, Q.-N. Yan, X.-M. Jiang and G.-C. Guo, *Chem. Mater.* 2021, **33**, 3729–3735.

[28] Z. Li, S. Zhang, Y. Guo, Z. Lin, J. Yao and Y. Wu, *Dalton Trans.* 2019, **48**, 6638–6644.

[29] X.-H. Li, Z.-H. Shi, M. Yang, W. Liu and S.-P. Guo, *Angew. Chem. Int. Ed.* 2022, **61**, e202115871.

[30] H.-J. Zhao, Y.-F. Zhang and L. Chen, *J. Am. Chem. Soc.* 2012, **134**, 1993–1995.

[31] C. Liu, S.-H. Zhou, Y. Xiao, C. Zhang, H. Lin and Y. Liu, *J. Mater. Chem. C* 2021, **9**, 15407–15414.

[32] B. Ji, A. Sarkar, K. Wu, A. Swindle and J. Wang, *Dalton Trans.* 2022, **51**, 4522–4531.

[33] J. Wang, H. Wu, H. Yu, Z. Hu, J. Wang and Y. Wu, *Adv. Opt. Mater.* 2022, **10**, 2102673.

[34] M.-M. Chen, Z. Ma, B.-X. Li, W.-B. Wei, X.-T. Wu, H. Lin and Q.-L. Zhu, *J. Mater. Chem. C* 2021, **9**, 1156–1163.

[35] Z.-T. Lu, Z.-D. Sun, Y. Chi, H.-G. Xue and S.-P. Guo, *Inorg. Chem.* 2019, **58**, 4619–4625.

[36] Z.-H. Shi, M. Yang, W.-D. Yao, W. Liu and S.-P. Guo, *Inorg. Chem.* 2021, **60**, 14390–14398.

[37] R. Ye, B.-W. Liu, X.-M. Jiang, J. Lu, H.-Y. Zeng and G.-C. Guo, *ACS Appl. Mater. Interfaces* 2020, **12**, 53950–53956.

[38] J. Zhou, L. Luo, Y. Chu, P. Wang, Z. Guo, X. Su and J. Li, *J. Alloys Compd.* 2022, **899**, 163366.

[39] S. L. Nguyen, J. I. Jang, J. B. Ketterson and M. G. Kanatzidis, *Inorg. Chem.* 2010, **49**, 9098–9100.

[40] C. Deng, X. Xu, Y. Hu, J. Guo, L.-M. Wu and L. Chen, *Inorg. Chem. Front.* 2025, **12**, 1437–1443.

[41] J. Zhou, H. Wang, J. Liu, X. Su, Y. Chu, J. Qu and X. Jiang, *Inorg. Chem. Front.* 2024, **11**, 2681–2689.

[42] H.-J. Zhao, *Z. Anorg. Allg. Chem.* 2016, **642**, 56–59.

[43] G. Yang, L.-H. Li, C. Wu, M. G. Humphrey and C. Zhang, *Inorg. Chem.* 2019, **58**, 12582–12589.

[44] H. Lin, Y.-Y. Li, M.-Y. Li, Z. Ma, L.-M. Wu, X.-T. Wu and Q.-L. Zhu, *J. Mater. Chem. C* 2019, **7**, 4638–4643.

[45] Y.-Z. Huang, H. Zhang, C.-S. Lin, W.-D. Cheng, Z. Guo and G.-L. Chai, *Cryst. Growth Des.* 2018, **18**, 1162–1167.

[46] Y. Xiao, M.-M. Chen, Y.-Y. Shen, P.-F. Liu, H. Lin and Y. Liu, *Inorg. Chem. Front.* 2021, **8**, 2835–2843.

[47] W.-F. Chen, B.-W. Liu, X.-M. Jiang and G.-C. Guo, *J. Alloys Compd.* 2022, **905**, 164090.
